# A New Drill Stock

**Published:** 1862-10

**Authors:** 


### A NEW DRILL STOCK.

WE have just received from Leslie's dental depot, of this city, a new drill socket, which is a very neat and convenient affair. It is designed to take the place of the drill ring. It consists of an ivory head, about one inch in diameter, in which plays a revolving socket, about an inch and a half long; this receives the end of the drill, which is rotated by the fingers in the usual manner. It is a good instrument, and can be used with great facility, and in some respects is preferable to the ring. We would suggest that at no place can dental goods be purchased to better advantage than at Leslie's Depot, corner of 4th and Race sts.

